# Behavioral and Transcriptomic Analyses in the Indoxacarb Response of a Non-Target Damselfly Species

**DOI:** 10.3390/insects15050367

**Published:** 2024-05-18

**Authors:** Bin Jiang, Wei Wang, Yu Yao, Haobo Zhang, Yongmei Zhang, Yang Sun

**Affiliations:** Provincial Key Laboratory for Conservation and Utilization of Important Biological Resources in Anhui, College of Life Sciences, Anhui Normal University, Wuhu 241000, China; wangwei_0430@163.com (W.W.); yao_yu123@163.com (Y.Y.); zhanghaobo@ahnu.edu.cn (H.Z.); zhym111111@163.com (Y.Z.)

**Keywords:** damselfly, indoxacarb, behavior, transcriptomic analysis

## Abstract

**Simple Summary:**

*Ischnura senegalensis*, a beneficial insect predator in the paddy fields, is perilously confronted with the survival challenges posed by insecticide application. In this study, we delved into the toxicity of indoxacarb on the larvae of *I. senegalensis*. Through behavioral experiments and transcriptome analyses, we uncovered that indoxacarb caused abnormal body movements and locomotory impairments, thereby posing a threat to larval survival. Notably, genes related to muscle function were significantly impacted. While lower concentrations of indoxacarb may be mitigated by the cytochrome P450 gene, higher concentrations significantly diminished the larvae’s sensory abilities and hampered toxicity degradation. Our findings highlight the importance of meticulously considering the impact of insecticides on non-target predatory insects before their widespread application.

**Abstract:**

*Ischnura senegalensis*, which widely spreads in paddy fields, has the potential to be used as a natural predator of insect pests. However, the application of insecticides in the field could pose a threat to the survival of *I. senegalensis*. Among these pesticides, indoxacarb, an oxadiazine insecticide, is renowned for its broad-spectrum efficacy against numerous insect pests. In this study, we examined the toxicity of indoxacarb towards the larvae of *I. senegalensis*. Behavioral experiments and transcriptome analyses were conducted under indoxacarb treatments. Results revealed that indoxacarb induced abnormal body gestures and significant locomotory impairments, which could ultimately reduce the survival rate of the larvae in their natural habitat. Moreover, transcriptome analyses indicated that genes related to muscle function were significantly affected. Interestingly, at lower concentrations of indoxacarb (0.004 mg/L), the larvae seem to detoxify the indoxacarb with the aid of the cytochrome P450 gene. However, under higher concentrations (0.4 mg/L), the sensory abilities of the larvae were significantly diminished, and they were unable to degrade the toxicity of indoxacarb. Our study underscores the importance of carefully evaluating the impact of insecticides on non-target predatory insects before their widespread application.

## 1. Introduction

Although people are developing ecologically friendly pest control methods, chemical control using pesticides is still the most effective and economical approach for pest management in many regions [1]. The application of pesticides has led to a significant reduction in insect biomass, particularly among nontarget insects, such as honeybees, ladybird beetles, and parasitoid wasps [2,3,4]. Further, pesticides could also cause sublethal damage to nontarget insects in various aspects, including abnormal orientating behavior [5], digestive physiology [6], reproduction [7], and gene expressions [3]. Therefore, greater attention must be paid to the toxic and sublethal effects of pesticides on beneficial nontarget insect communities [8].

Odonates, a group of beneficial nontarget insects, are widely distributed both in natural habitats and in areas heavily influenced by human activities (e.g., rice fields and city parks). Their particular sensitivity to water quality makes them crucial bioindicators of ecosystem health [9,10]. Odonates are typically important predators of disease vectors (e.g., mosquitos) and agricultural pests (e.g., rice leaf rollers and brown planthoppers) [11]. Further, the larvae of odonates play pivotal roles as intermediate or top predators in many aquatic ecosystems [12,13]. Research has shown that pesticide concentrations detected in surface water are negatively correlated with the abundance of odonates [14]. The ecological side effect of pesticides on insect communities, especially on damselflies, may be underestimated [15]. To fully understand this, more specific research on the sublethal effects of pesticides on damselflies should be carried out.

The sublethal effects of various widely used pesticides on beneficial arthropods have been examined. Among those widely used pesticides, indoxacarb is an oxadiazine insecticide used to control lepidopteran pests, such as rice leaf folders [16]. In Europe, indoxacarb is registered and applied to crops like maize, sweet corn, and lettuce [17]. In China, hundreds of commercial indoxacarb products are registered specifically for pest control in rice [18]. Indoxacarb works by inhibiting sodium ion entry into nerve cells [19] and is recognized as highly toxic to aquatic invertebrates with prolonged effects [20]. For instance, chronic exposure to indoxacarb has been found to hinder the normal growth and development of non-biting midges, while short-term exposure alters the activity of detoxification enzymes [20]. Due to its neural toxicity, the application of indoxacarb has been shown to reduce locomotory activities among maize weevil populations [21]. Furthermore, prolonged exposure to indoxacarb causes abnormal behaviors (e.g., disorientation and ataxia) in the Eastern Subterranean termites [22]. Such behavioral alterations can decrease the survival ability of both target and nontarget insects. Further, transcriptomic analysis was also widely used to examine the impact of indoxacarb. In German cockroaches, after six generations of selection, 15 genes out of 20 top differently expressed genes (DEGs) are related to Cytochrome P450, meaning that cockroaches could rapidly evolve indoxacarb resistance [23]. In red imported fire ant (*Solenopsis invicta*), metabolic pathways, fatty acid metabolism, and insulin signaling pathways are the pathways that comprise the maximum highly regulated unigenes under indoxacarb treatment [24].

Indoxacarb exhibits varying toxicity towards different insect species. Some pests develop resistance to indoxacarb due to the overexpression of detoxification enzyme families, such as in the case of *Spodoptera litura* [25] and *Blattella germanica* [23]. Conversely, non-target insects (e.g., predatory insects) have demonstrated high sensitivity to indoxacarb [26,27]. Therefore, when considering nontarget insects, it is crucial to clearly assess the safety of using indoxacarb in specific environments. Among odonates, *Ischnura senegalensis* is globally distributed, with a widespread presence in rice fields [28,29]. This species serves as an important predator in rice fields [30] and is a suitable bio-indicator for assessing the safety of pesticides [31]. Therefore, it is crucial to investigate whether indoxacarb is safe for predatory insects when applied in rice fields. Further, as a neurotoxic pesticide, indoxacarb will inevitably impose an impact on the locomotion of larvae. To address this, we employed *I. senegalensis* larvae as bio-indicators to assess the toxicity of indoxacarb. Sublethal concentrations of indoxacarb were applied, and the larval behavioral responses were recorded. Transcriptome analyses were conducted to investigate whether the larvae could respond to indoxacarb with certain detoxification enzymes.

## 2. Materials and Methods

### 2.1. Sample Collection and Larvae Rearing

Mated female damselflies were collected from the paddy fields along the Yangzi River in Wuhu, Anhui province of China in mid-July of 2021–2022. The adult females were placed individually in transparent plastic containers to lay eggs on moist filter paper. The eggs were submerged in water and incubated at 35 ± 2 °C (photoperiod: L:D = 13:11) until hatching occurred within 7–8 days. Subsequently, the larvae were reared in a large plastic container to acclimate to the lab environment until they reached the last and penultimate instars for our following experiments. The larvae were fed regularly and *ad libitum* with *Artemia* and water fleas.

### 2.2. Behavioral Experiment with Indoxacarb Treatments

Larvae were individually placed in new 25 mL transparent plastic containers with a small piece of net for perching. According to our pretrials, the recommended spraying dosage [32,33], and indoxacarb dissipation rate in the rice field [34,35], we set five gradient concentrations (0.008 mg/L, 0.04 mg/L, 0.2 mg/L, 1 mg/L, 5 mg/L), a control (CK) and solvent control (AC). A solvent control was tested because we first dissolved 10 mg of indoxacarb in 10 µL of acetone before dispersing it in aged tap water. Fifteen larvae were used for each treatment.

Larvae were exposed to indoxacarb for 72 h. Behaviors were classified into two categories, namely body gesture and moving ability. Body gestures were ranked according to larvae’s ability to stay motionless, which is the behavior ambush predators usually do (so-called sit and wait for prey). If their muscles were paralyzed, it would be hard for them to cling to substances. For moving abilities, we recorded their responses when the larvae get stimulated with tweezers. Larvae usually escape from disturbance; however, they might become unresponsive from disturbance after being poisoned by indoxacarb. The body status and larval movement were recorded for 5 min after 24 h, 48 h, and 72 h for each treatment. Behavior recording details and ranking of behaviors were according to Table 1. The chi-square test of independence was used to determine the independence between the observed behaviors and treatments in R 4.3.0. Furthermore, the survival rate was calculated for each treatment.

### 2.3. Transcriptome Analyses on Larvae with Indoxacarb Treatments

Based on the behavioral experiment, there were no dead larvae under 0.04 mg/L indoxacarb treatment. Therefore, three low-lethal concentrations (0.004 mg/L, 0.04 mg/L, 0.4 mg/L) were selected in the transcriptome analyses. In the following analysis, we used L (low concentration), M (median concentration), and H (high concentration), representing the above three concentrations, respectively. A control and a solvent control were also set in the transcriptome analyses experiment. Another group of mated females was also collected for transcriptome analysis from Wuhu, Anhui Province, and larvae were raised from the egg stage as for the behavioral experiments. For each treatment, 12 larvae were used during the experiment. After 72 h indoxacarb treatments, larvae were collected, put into liquid nitrogen, and stored in a −80 °C refrigerator.

#### 2.3.1. Total RNA Extraction and cDNA Library Construction

For each treatment, six larvae were randomly selected for RNA extraction, and one larva represented one replication for RNAseq analyses. Total RNA was extracted using TRIzol reagent (Invitrogen, Carlsbad, CA, USA). RNA quality and concentration were checked on 1% agarose gels and Agilent 2100 RNA Nano 6000 Assay Kit (Agilent Technologies, Santa Clara, CA, USA). The cDNA library was constructed following the manufacturer’s instructions for the NEB Next Ultra Directional RNA LibraryPrep Kit for Illumina (NEB, Ipswich, MA, USA). In brief, the mRNA was enriched by Oligo (dT) magnetic beads and then fragmented using a fragmentation buffer. These fragments were used to synthesize the first-strand cDNA and the second cDNA. End-repair was performed on the synthesized cDNA, followed by dA-tail and adaptor ligation. Standard AMPure XP (Beckman Coulter, Inc., Brea, CA, USA) was used to select DNA fragments of preferentially 150–200 bp in length. The constructed cDNA libraries were sequenced using an Illumina HiSeq platform with the paired-end reads in the Core PE150.

#### 2.3.2. Reference Genome-Based Reads Mapping and Annotation

Low-quality raw reads with Phred Quality Score ≤ 19 and unknown nucleotides ≥ 5% were removed using fastp [36]. Due to the unavailability of the genome of *I. senegalensis*, clean reads filtered from raw reads were mapped to the same genus species, *Ischnura elegans*, genome (Zenodo, DOI: 10.5281/zenodo.4679993) [37] using HiSAT2 [38]. Genes were annotated by BLASTn with a cut-off E-value of 10^−5^ against non-redundant nucleotide sequence (NT). BlastP with a cut-off E-value of 10^−5^ was used when genes were compared against the UniProt database and the Homologous protein family database (Pfam). Gene Ontology database (GO) annotations were obtained by Blast2GO [39]. Kyoto Encyclopedia of Genes and Genomes (KEGG) pathways were analyzed by KOBAS v3.0 software to test the statistical enrichment of differential expression genes [40].

#### 2.3.3. Analysis of Differentially Expressed Genes (DEGs)

We calculated gene expression values by the read/fragments per kilobase of exon per million fragments mapped (RPKM/FPKM) formula using Cuffdiff (http://cufflinks.cbcb.umd.edu/, accessed on 10 May 2022) [41,42]. Differential gene expression between AC and different concentrations of indoxacarb treatments (H vs. AC, M vs. AC, L vs. AC) was identified using DEseq [43]. The false discovery rate (FDR) was used to determine the threshold P-value in multiple tests. Only genes with |log2FoldChang| ≥ 1 and q < 0.05 (adjusted *p*-value) were considered as significantly different and used for subsequent analysis. GO annotation and KEGG enrichment analysis were carried out for DEGs.

#### 2.3.4. Quantitative Real-Time PCR (RT-qPCR) Analysis

To confirm the DEG results from Illumina sequencing, seven genes related to muscle function and behavior responses were selected, and beta-actin was selected as a reference gene (Table 2). Their specific primers were designed with Primer3web v4.1.0 (https://primer3.ut.ee/, accessed on 13 December 2022) [44]. The reaction system of qPCR contained 10 µL SYBR Premix Ex Taq (2×), 0.8 µL × 10 μM × 2 forward and backward primers, 1 µL cDNA templates, and 7.4 µL ddH_2_O. The recycling parameters of PCR were 95 °C for 30 s, 40 cycles with 95 °C for 5 s, and 60 °C for 20 s. For qPCR analysis, three biological replicates were used. The gene expression level was calculated by the 2^−ΔΔCt^ method [45]. The Pearson Correlation Coefficient between qPCR data and RNAseq data were calculated using Excel LTSC professional plus 2021.

## 3. Results

### 3.1. Behavior Changes under Indoxacarb Treatments

**Figure 1:**
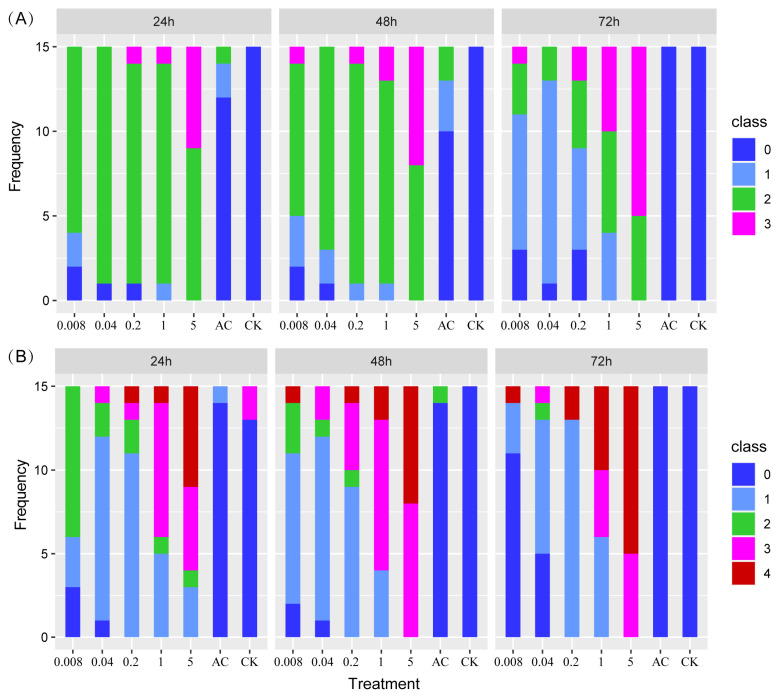
Body gesture (**A**) and moving ability (**B**) of larvae during indoxacarb treatments. Different colors represent different rankings of the behavior index. For the ranking, please refer to Table 1.

**Figure 2:**
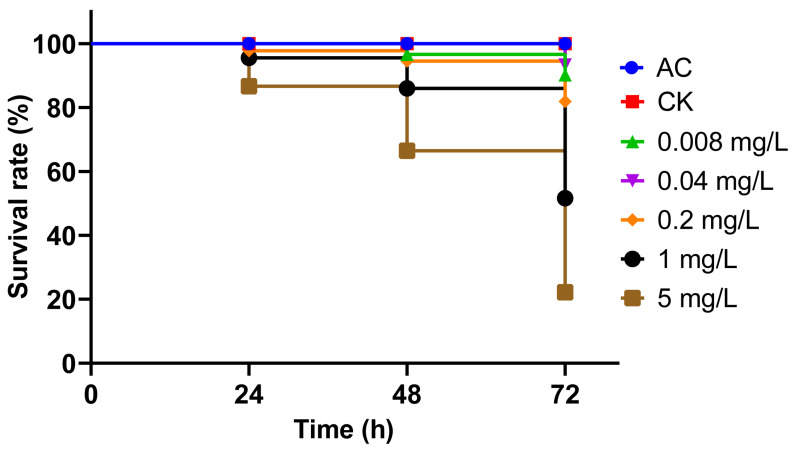
Survival rate of *Ischnura senegalensis* larvae in solvent control (AC), control (CK), and different concentrations of Indoxacarb treatments (0.008 mg/L, 0.04 mg/L, 0.2 mg/L, 1 mg/L, 5 mg/L) after 72 h.

According to the observed status and moving ability of the larvae, we categorized their behavior into distinct classes (Table 1). Both body gesture (χ^2^ = 315, df = 18, *p* < 0.01) and moving ability (χ^2^ = 413, df = 24, *p* < 0.01) were significantly related to applied treatments. Furthermore, these two behaviors were significantly related to the duration of treatment (body gesture: χ^2^ = 51, df = 6, *p* < 0.01; moving ability: χ^2^ = 27, df = 8, *p* < 0.01). As the concentration of indoxacarb increased and the duration of treatment prolonged, the impact of indoxacarb on both of the above larval behaviors became more severe (Figure 1). The survival rate of each treatment comparing AC is shown in Figure 2.

### 3.2. Transcriptome Analysis of Behavior Defection

#### 3.2.1. RNA Sequencing and Assembled Unigenes

**Figure 3:**
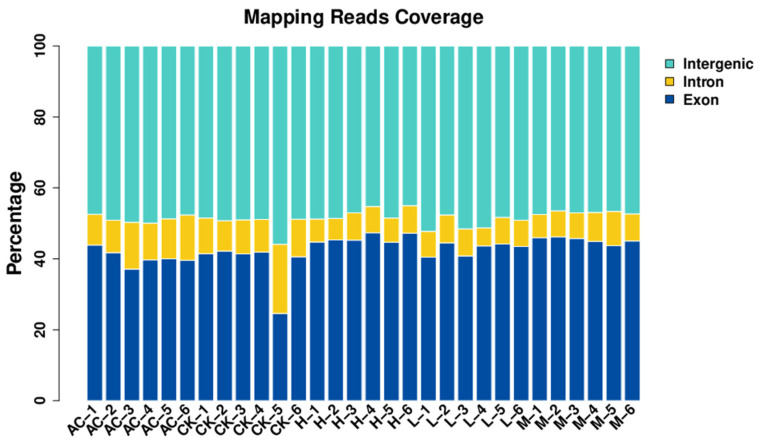
The percentage of different regions from the reads mapping to the reference genome *Ischnura elegans*. Samples of control (CK 1-6), solvent control (AC1-6), and different concentrations (L1-6, M1-6, and H1-6) were shown in the x-axis.

In this study, each sample yielded, on average, 45.4 million clean reads, amounting to a total of 84.9 Gb of raw reads. In total, 30 RNA-Seq libraries were established, with the Q20 and Q30 of each library exceeding 96.8% and 91.5%, respectively. A small proportion mapped to multiple locations, averaging 1.3%. When aligned to the reference genome of *Ischnura elegans*, the average mapping rate of our data is 57.3%. Most of the sequences aligned with the reference genome mapped to exon, averaging 42.6%, while only 8.8% were mapped to intron on average (Figure 3).

#### 3.2.2. GO and KEGG Enrichment Analyses of DEGs

**Figure 4:**
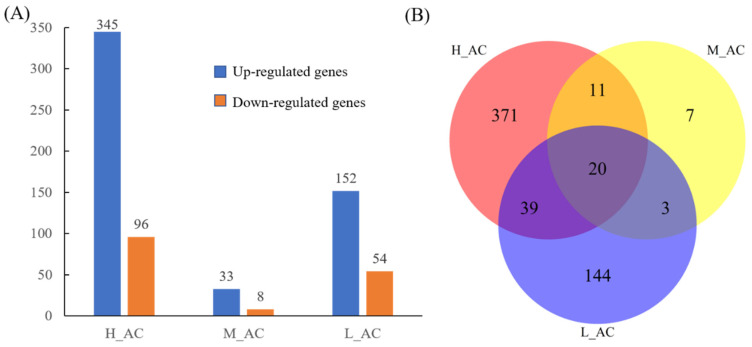
(**A**) The number of up-regulated and down-regulated genes in comparisons between different indoxacarb treatments (H, M, L) and solvent control (AC); (**B**) Venn diagram of the differentially-expressed genes in comparisons between different indoxacarb treatments (H, M, L) and solvent control (AC). The numbers in the histogram and each circle represent the DEG numbers.

Gene expression levels were calculated as FPKM values. When comparing the analysis between water (CK) and solvent controls (AC), only one DEG was found. Between AC and different indoxacarb treatments, DEGs were shown in Figure 4A (for DEGs from each comparison see Supplementary Material). Treated with different concentrations of indoxacarb, 20 genes were commonly identified, which are all up-regulated (Table 3).

**Figure 5:**
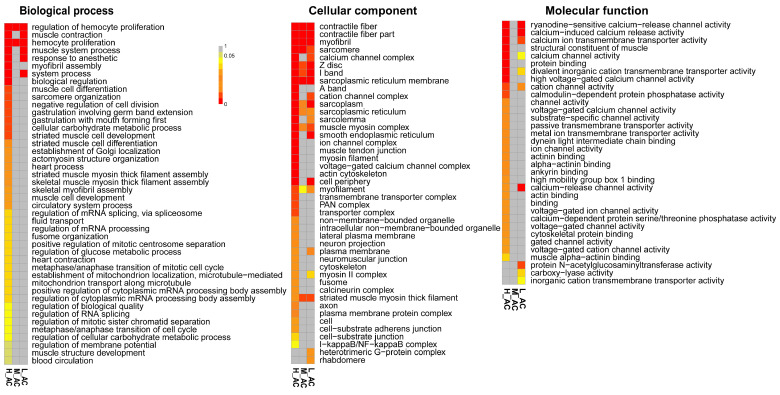
The enrichment GO terms of DEGs in three major categories. Different colors indicate the q values of the subcategories.

GO enrichment analysis was performed to elucidate the functions of DEGs. Among categories of biological process, cellular component, and molecular function, a total of 44, 42, and 31 subcategories were identified as being highly regulated (q < 0.05) (Figure 5). Notably, many of those subcategories are associated with the proper functioning of muscles. For instance, within the biological process category, five of the top 10 subcategories are directly related to muscle, including muscle contraction, muscle system process, myofibril assembly, muscle cell differentiation, and sarcomere organization.

**Figure 6:**
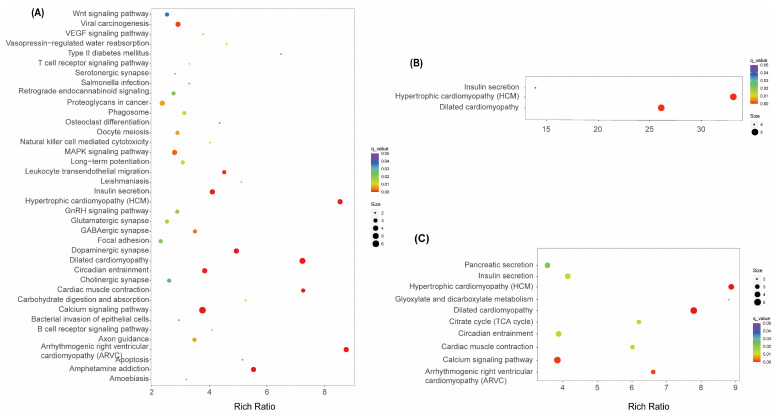
KEGG enrichment of the KEGG pathways for DEGs comparing indoxacarb treatments and solvent control (AC). DEGs by comparing (**A**) H and AC, (**B**) M and AC, and (**C**) L and AC.

To identify enriched biological pathways in response to indoxacarb treatment, KEGG enrichment analyses were conducted to assess the function of DEGs. When comparing the high concentration of indoxacarb treatment with the solvent control (H vs. AC), 38 biological pathways were found to be enriched with DEGs (Figure 6). Three biological pathways and ten pathways were enriched in M vs. AC and L vs. AC, respectively. Across all three comparisons, the shared pathways were associated with muscle disease (Hypertrophic cardiomyopathy and Dilated cardiomyopathy) and metabolic disorder (Insulin secretion). In both H vs. AC and L vs. AC, additional pathways related to the calcium signaling pathway and circadian entrainment were also discovered. In H vs. AC, pathways were discovered to be closely related to body damage repair (Apoptosis, Phagosome, Osteoclast differentiation, T cell receptor signaling pathway, and Natural killer cell-mediated cytotoxicity) and neural signal transduction (pathways involving Synapse, Axon guidance, Long-term potentiation, Amphetamine addiction, and Retrograde endocannabinoid signaling).

#### 3.2.3. DEGs Validation with RT-qPCR

**Figure 7:**
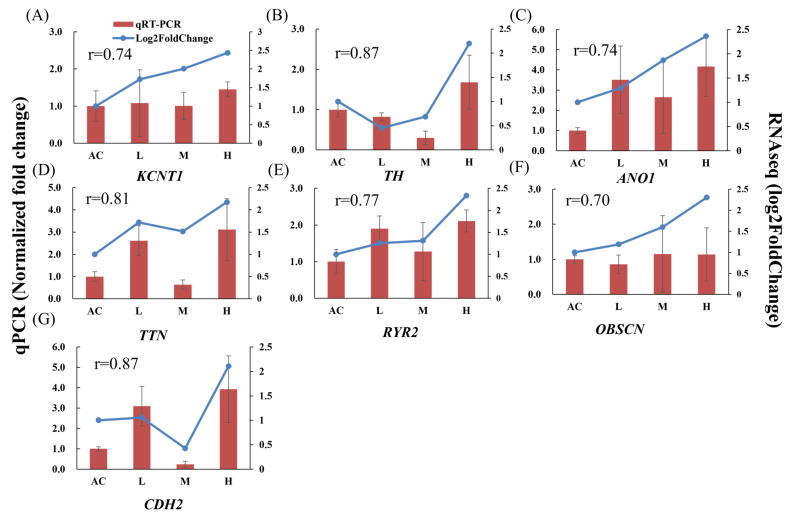
Validation of expressions of chosen DEGs compared with transcriptome using qPCR. The correlation coefficient was shown in each figure. (**A**–**G**) are the selected genes for qPCR and gene information refers to Table 2.

Seven genes, namely *KCNT1*, *TH*, *TTN*, *RYR2*, *ANO1*, *OBSCN*, and *CDH2*, were chosen for qPCR validation of the RNA-seq data (Figure 7). The RT-qPCR results exhibited consistent expression trends with the transcriptome sequencing (correlation coefficient all > 0.70). Among those genes, *TTN* and *OBSCN* encode proteins that are integral components of muscle. *KCNT1*, *TH*, *RYR2*, *ANO1*, and *CDH2* are associated with ion channel and neural signal transduction.

## 4. Discussion

In agricultural practices, beneficial nontarget insect communities are easily affected. Indoxacarb is widely used for managing a diverse range of lepidopteran pests, such as *Spodoptera* sp., *Plutella* sp., and *Laspeyresia* sp. [46,47]. In this study, we evaluated the impact of indoxacarb on the behavior of *I. senegalensis* larvae. Our results indicated that under indoxacarb treatments (0.2 mg/L, 1 mg/L, and 5 mg/L), *I. senegalensis* exhibited altered behavior, displaying abnormal body gestures and significant locomotory impairments. Dosages below 0.04 mg/L appeared to be safe for the last and penultimate instar larvae. However, it remains unclear whether the larvae in earlier instars can survive from this dosage, and further experiments are needed to clarify this. Indoxacarb is a neurotoxic pesticide that typically induces strong feeding inhibition in target pests [48]. Our findings demonstrated that larval *I. senegalensis* exhibited similar neurotoxic symptoms, including convulsions in the leg and body, as well as pseudoparalysis. After 72 h of indoxacarb treatment, the larvae’s feeding behavior was greatly suppressed due to their body convulsions. Abnormal behavior in larvae can lead to high mortality rates in the wild. Paralyzed and floating larvae are easily preyed upon and may starve to death [49].

Indoxacarb should be applied in an environmentally safe dosage. Based on residual dynamics experiments conducted in a paddy field, when indoxacarb is sprayed at an active ingredient concentration of 36g a.i./ha, the initial concentration of indoxacarb in the water (sampling 2 h after spraying) ranges from 0.006 to 0.016 mg/kg [35]. Combined with our results, this concentration might have little impact on final and penultimate instar damselfly larvae. However, their toxicity on early instars should also be considered. However, when the spraying concentration increases to 67.5 g a.i./ha, the initial concentration of indoxacarb in the water rises to approximately 0.27 to 0.4 mg/kg [34,50], exceeding the toxic concentration threshold observed in our experiments. Indoxacarb can cause adverse effects on non-target insects. Although indoxacarb is less harmful to predatory Hemipterans compared to other broad-spectrum insecticides (such as pyrethroid and organophosphate), it still reduces the Hemipteran population in the field [51]. For example, in the predatory stink bug (*Podisus distinctus*), indoxacarb has been shown to reduce respiration, inhibit food consumption, and cause hyperexcitation [48]. While in our damselfly larvae, KEGG analysis indicated that the insulin secretion pathway was significantly influenced in all H, M, and L treatments. Further, three other pathways in L treatment (Glyoxylate and dicarboxylate metabolism, Pancreatic secretion, and Citrate cycle) and one other pathway in H treatment (Carbohydrate digestion and absorption) were also influenced. This indicates that indoxacarb also hindered metabolism in *I. senegalensis* larvae, analogous to its effect on stink bugs. Therefore, to safeguard beneficial predatory insects, including damselflies, utmost caution must be exercised in the field application of indoxacarb.

Transcriptome analyses were also conducted to understand the mechanism of indoxacarb toxicity. Our results suggested that genes related to muscle structure and proper function were significantly affected. Among the 20 shared upregulated genes across the three treatments (L, M, and H) (Table 3), *RYR2*, *KCNT1*, *ANO1*, and *KCNKN*, were particularly noteworthy, as they are all associated with ion channels in biomembranes (Ca^2+^ and K^+^ channels). Ca^2+^ plays a central role in muscle excitation-contraction coupling, and both Ca^2+^ and K^+^ are crucial ionic components for membrane currents in muscle fibers [52]. Additionally, *JPH* genes (junctophilin), pivotal regulators of Ca^2+^ dynamics at the endoplasmic reticulum–plasma membrane junctions, were found to be upregulated [53]. Notably, variations in the expression levels of these *JPH* genes have been associated with muscular deficiencies and neuronal changes in *Drosophila* [54]. The energy metabolic-related gene, NADH dehydrogenase (ubiquinone) Fe-S protein 1 (*NDUFS1*), was also upregulated, which is one of the core subunits forming mitochondrial complex I [55]. These shared upregulated genes could potentially explain the abnormal behavior in damselfly larvae treated with indoxacarb. Further, in L treatment, only GO terms of muscle contraction, muscle system process, and response to anesthetic were significantly affected (Figure 5). Those terms are all related to the proper movement of larvae. While in H treatment, GO terms associated with ion channel function, protein binding, myofibril assembly, muscle cell differentiation, sarcomere organization, and striated muscle cell development were heavily influenced. Those terms will impair the formation of muscles and thus impact the proper function of muscles. These results indicate that a high concentration of indoxacarb might induce unreversible harm to larval muscles.

At the low concentration treatment (0.004 mg/L), the cytochrome P450 genes were upregulated. Cytochrome P450, which catalyzes the metabolism of exogenous compounds, plays an important role in detoxifying insecticides [56]. However, at the medium concentration treatment (0.04 mg/L), no detoxification-related DEG was observed. Conversely, at the high concentration treatment (0.4 mg/L), the cytochrome P450 gene was downregulated. These observations suggest that these two concentrations of indoxacarb (medium and high) exceed the detoxification capabilities of *I. senegalensis* larvae. Furthermore, the median concentration treatment exhibited the lowest number of DEGs compared to both low and high concentrations. It appears that an indoxacarb shock may occur in larvae when the concentration of indoxacarb increases from 0.004 mg/L to 0.04 mg/L. Under this concentration, larvae might be unable to fight against the toxicity of indoxacarb at all. Under high-concentration treatments, the down-regulated genes were related to the pathway of body sensor, lipid metabolism, mRNA processing, and protein interaction. For instance, cryptochrome 1 (*CRY1*) and nuclear receptor subfamily 1 group D member 3 (*NR1D3*) were down-regulated, both of which are linked to circadian rhythm. Additionally, glutaredoxin domain-containing cysteine-rich protein 1 (*GRXCR1*), which is involved in the sensory processing of sound and olfactory signals, was also severely affected. Those alterations could explain the slow response observed in the larval behavioral experiments. Those slow responses may also connect to the defective nervous system. Indoxacarb, working as a neurotoxic pesticide, also imposed an influence on DEG terms of voltage-gated calcium channel complex, neuron projection, neuromuscular junction, axon, and calcineurin complex. Additionally, the neuronal acetylcholine receptor gene was down-regulated in L treatment, and acetylcholinesterase was up-regulated in H treatment. Moreover, the DNA damage sensor (*BUB1*) and the DNA-Dependent Protein Kinase Catalytic Subunit (*PRKDC*), which are involved in DNA repair, were also down-regulated. These findings suggested that, under 0.4 mg/L indoxacarb treatment, larvae might lose their ability to sense their surroundings.

In our study, the toxicity of indoxacarb on *I. senegalensis* was examined. Indoxacarb exhibited neurotoxicity on the larvae, and their behaviors became impaired under treatments with concentrations exceeding 0.2 mg/L. By analyzing the transcriptome of the larvae exposed to indoxacarb, numerous genes related to the muscle and nervous system were detected. Therefore, the application of indoxacarb should be careful and strictly examined. Furthermore, *I. senegalensis*, as one of the crucial intermediate predators in the paddy fields, plays a vital role in maintaining the ecological balance of the field. Our experiments suggest that damselfly larvae could be a suitable model for testing the safety of pesticides. Widespread applications of pesticides pose a significant threat to such beneficial predators in agricultural ecosystems, potentially further leading to the destruction of a healthy ecosystem. Although some insecticides are environmentally friendly or have low toxicity to mammals, their safety to predatory insects must also be considered before the widespread application of insecticides. Moreover, considering the different environmental conditions between the laboratory and the wild, pesticide impacts in the wild condition should also be carefully examined in the future.

## Figures and Tables

**Table 1:** The description of two behavior indexes and their ranking of indoxacarb impact.

Behavior Index	Description	Ranking of Impact
Body gesture	Sit on the net or wall of the container	0
	Clinging to the net loosely by not all legs	1
	Upside down or floating without clinging to the net	2
	Dead without clinging	3
Moving ability	Behave normally and swim quickly	0
	Behave normally and respond slowly with one touch	1
	Body convulsion and respond slowly with one touch	2
	Little response after several touches	3
	No response with touches (dead)	4

**Table 2:** RT-qPCR primers were used to validate DEG results responding to indoxacarb in *Ischnura senegalensis*.

Gene	Forward Primer (5′-3′)	Backward Primer (5′-3′)
*beta-actin*	CTTCCTGTGCACGATGGA	ACCGTATGCAGAAGGAGA
*Potassium Sodium-Activated Channel Subfamily T Member 1 (KCNT1)*	ATTCCCTCGAGACCCAGA	GAATGGCTCAACGTCACC
*Anoctamin 1 (ANO1)*	GCGGTAGAAAGTAAGGAA	AAGAGAGTCGGGAGAATA
*Ryanodine Receptor 2 (RYR2)*	GGAAACTGAGGAATGACA	AGGAAAGGAAACAAGACA
*Titin (TTN)*	CTTACGCCAGTGAACGAT	CCACAGCCAGAAGAACCT
*Tyrosine Hydroxylase (TH)*	CATCAAAGACAGAGGGCG	GTCAGCGAGAAGCGAAAC
*Obscurin (OBSCN)*	GTTTTCTACACCCTCCTG	AACTTTTGTCGTCCCCTA
*Cadherin 2 (CDH2)*	CCCTGATCTTGAACGTCT	TCTTCCTCCTCGTCATCC

**Table 3:** The shared 20 DEGs across all three indoxacarb treatments (L, M, and H).

Gene	Gene Description
*RYR2_1*	Ryanodine receptor
*TTN_1*	Titin
*KCNT1*	Potassium channel subfamily T member 1
*TRAPP*	Transport protein particle component
*JPH*	Junctophilin-1
*Methyltrans_Mon*	Virus-capping methyltransferase.
*MIF4G*	MIF4G domain
*PH*	Oxysterol-binding protein 1
*zf-C3HC4*	Zinc finger, C3HC4 type (RING finger)
*RYR2_2*	Ryanodine receptor
*DUF4775*	Domain of unknown function (DUF4775)
*ANO1*	Anoctamin-1
*TTN_2*	Titin
*Hamartin*	Hamartin protein
*CTF_NFI*	CTF/NF-I family transcription modulation region
*REL*	c-Rel proto-oncogene protein
*DDE_Tnp_1_7*	Transposase IS4
*Drf_FH1*	Formin Homology Region 1
*NDUFS1*	NADH-ubiquinone oxidoreductase 75 kDa subunit
*KCNKN*	TWiK family of potassium channels protein 7

## Data Availability

The raw reads were submitted to the Short Read Archive (SRA) and BioProject accession number PRJNA1097672 (https://www.ncbi.nlm.nih.gov/bioproject/PRJNA1097672/, accessed on 8 April 2024).

## Supplementary Materials

Supplementary materials for DEGs from different comparisons (L vs. AC, M vs. AC, and H vs. AC) can be downloaded at: https://www.mdpi.com/article/10.3390/insects15050367/s1.
